# Biosynthetic Gene Cluster for the Cladoniamides, Bis-Indoles with a Rearranged Scaffold

**DOI:** 10.1371/journal.pone.0023694

**Published:** 2011-08-18

**Authors:** Katherine S. Ryan

**Affiliations:** Department of Chemistry, The University of British Columbia, Vancouver, British Columbia, Canada; Deutsches Krebsforschungszentrum, Germany

## Abstract

The cladoniamides are bis-indole alkaloids isolated from *Streptomyces uncialis*, a lichen-associated actinomycete strain. The cladoniamides have an unusual, indenotryptoline structure rarely observed among bis-indole alkaloids. I report here the isolation, sequencing, and annotation of the cladoniamide biosynthetic gene cluster and compare it to the recently published gene cluster for BE-54017, a closely related indenotryptoline natural product. The cladoniamide gene cluster differs from the BE-54017 gene cluster in gene organization and in the absence of one *N-*methyltransferase gene but otherwise contains close homologs to all genes in the BE-54017 cluster. Both gene clusters encode enzymes needed for the construction of an indolocarbazole core, as well as flavin-dependent enzymes putatively involved in generating the indenotryptoline scaffold from an indolocarbazole. These two bis-indolic gene clusters exemplify the diversity of biosynthetic routes that begin from the oxidative dimerization of two molecules of l-tryptophan, highlight enzymes for further study, and provide new opportunities for combinatorial engineering.

## Introduction

**Figure 1:**
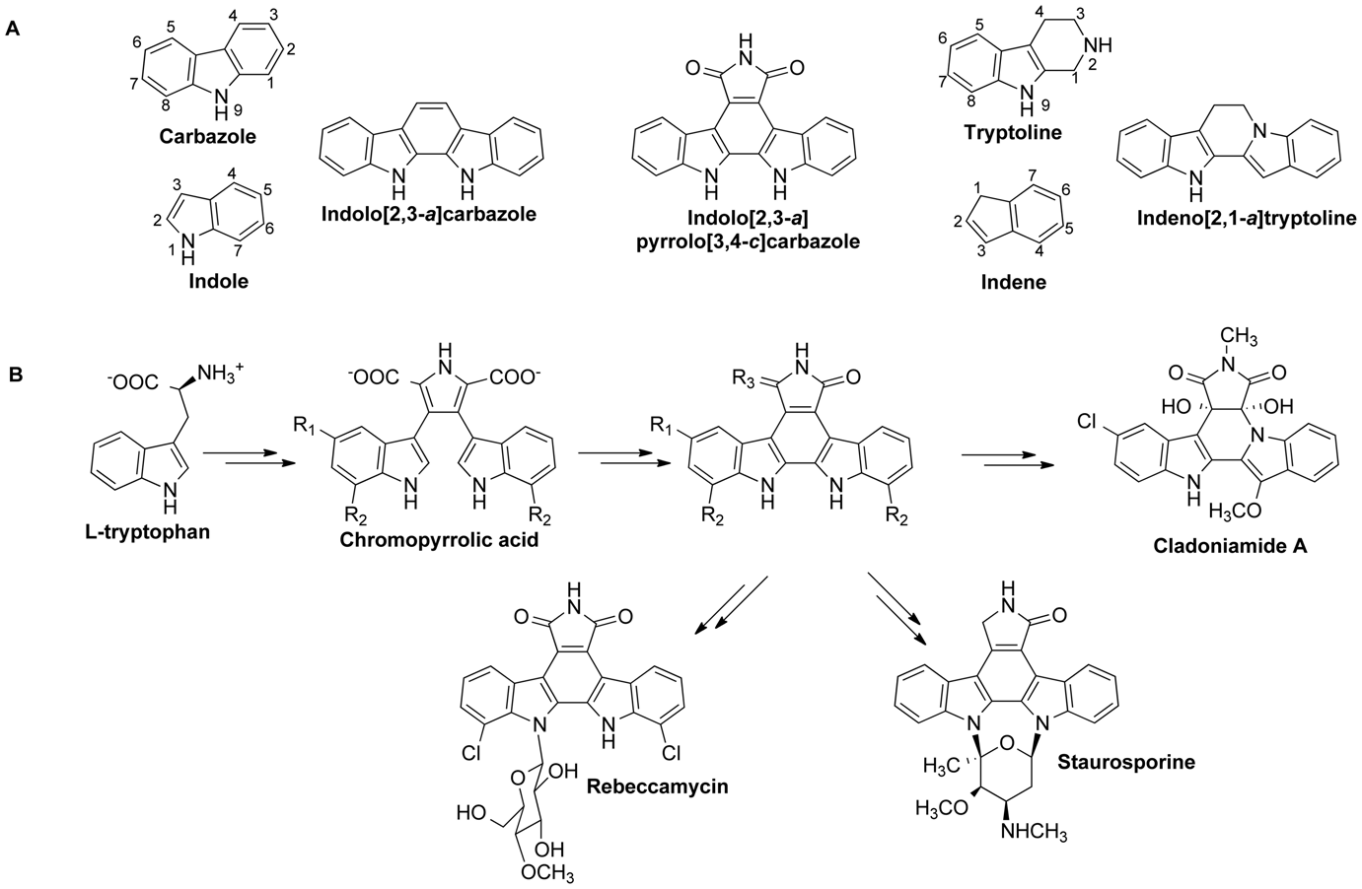
Bis-indole biosynthesis. (A) Chemical structures described in the text. (B) Overall biosynthetic pathways to rebeccamycin, staurosporine, and cladoniamide A. R_1_ = H, R_2_ = Cl, R_3_ = O in rebeccamycin pathway; R_1_,R_2_ = H, R_3_ = 2H in the staurosporine pathway; R_1_ = Cl, R_2_ = H, R_3_ = O in the cladoniamide A pathway.

*Streptomyces uncialis* is an actinomycete bacterial strain isolated with the lichen *Cladonia uncialis* near the Pitt River in British Columbia. This bacterial strain is the source of the enediyne uncialamycin [1], [2], and the cladoniamides, a series of bis-indole alkaloids [3]. The cladoniamides are unusual among bis-indole natural products: most bis-indoles have an indolocarbazole structure [4], whereas the cladoniamides have a rarely observed indenotryptoline structure (Figure 1). The commonly observed indolocarbazole structure is found in a number of important molecules, including rebeccamycin, analogs of which are DNA-topoisomerase I inhibitors [5], [6], and staurosporine, a kinase inhibitor [7], [8]. Compounds related to rebeccamycin and staurosporine, including becatecarin and UCN-01, have been in multiple clinical trials against cancers (http://clinicaltrials.gov/ct2/home). The indolocarbazole core in rebeccamycin, staurosporine, and related natural product molecules derives biosynthetically from the face-to-face dimerization of two molecules of l-tryptophan [4], [9], [10], [11], [12]. By contrast, the indenotryptoline scaffold found in the cladoniamides cannot be derived in this manner, given that one indole ring is ‘flipped’ relative to the other in the final structure. Williams et al. have postulated that the cladoniamides are biosynthetically generated from the enzymatic degradation and rearrangement of an indolocarbazole intermediate [3].

**Figure 2:**
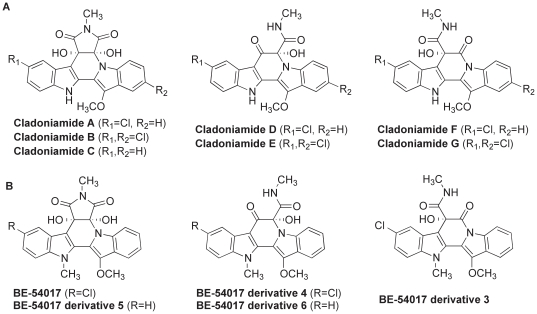
Structures of cladoniamides and BE-54017 and its derivatives. (A) Cladoniamides from *Streptomyces uncialis* and (B) BE-54017 and its derivatives. Derivatives of BE-54017 are labelled to be consistent with the labels used by Chang and Brady [13]; the compounds are currently unnamed. The major cladoniamide from *Streptomyces uncialis* is cladoniamide A [3]; the major metabolite from the heterologous expression of the BE-54017 gene cluster in *Streptomyces albus* is BE-54017 [13].

Here the details of the cladoniamide (*cla*) gene cluster from *Streptomyces uncialis* are reported. The *cla* cluster is compared to the recently published BE-54017 (*abe*) gene cluster from environmental DNA [13]. Chemically, BE-54017 and its derivatives are identical to the cladoniamides, except for the presence of one additional *N-*methyl group and the lack of di-chloro derivatives (Figure 2). The BE-54017 and cladoniamide gene clusters differ in overall organization of genes and in the absence of one *N-*methyltransferase gene in the cladoniamide gene cluster. However, the gene clusters share other major features, including encoding enzymes needed for the construction of an indolocarbazole core and flavin-dependent enzymes putatively involved in the oxidative chemistry that facilitates formation of the indenotryptoline structure. The heterologous expression and transposon mutagenesis study reported with the BE-54017 gene cluster thus provides substantial experimental insight into the biosynthetic construction of not only BE-54017 but also the cladoniamides. The elucidation of both gene clusters supports the hypothesis that indenotryptoline cores are biosynthetically derived from the oxidative rearrangement of an indolocarbazole precursor. The availability of both gene clusters should enable further studies in combinatorial engineering of bis-indoles and exploration of the biochemistry and structural biology of the new enzymes identified in these studies.

## Results and Discussion

### Identification of the cladoniamide gene cluster

The cladoniamide producer *Streptomyces uncialis* [1], [3] was cultivated in YEME media and genomic DNA was isolated. Degenerate primers specific for conserved regions of the indolocarbazole gene *rebC* [10] and its homologs (Figure S1) [9], [14], [15], [16], [17] were used in the polymerase chain reaction to amplify a portion of the corresponding *rebC* homolog from *Streptomyces uncialis*. The sequence of this fragment was used for design of specific primers for screening of a cosmid library of *Streptomyces uncialis* genomic DNA by PCR. Five positive cosmids were identified, and following end sequencing, one cosmid predicted to contain the complete gene cluster was fully sequenced (see Materials and Methods).

The completely sequenced cosmid has an insert of ∼37.6 kilobasepairs (kbp). The cladoniamide gene cluster consists of 13 open reading frames (ORFs), spanning ∼20.7 kbp. These ORFs are in the central region of the cosmid, with ∼9.6 kbp of additional sequence upstream and ∼7.3 kbp of additional sequence downstream of the cluster.

### Similarity of the cladoniamide and BE-54017 gene clusters

My annotation of individual genes in the cladoniamide (*cla*) cluster matches well with the independently reported BE-54017 (*abe*) cluster [13]. Each gene in the *cla* gene cluster has a homolog in the *abe* gene cluster, and encoded proteins are 46–74% identical on the amino acid level (Table 1). The *abe* cluster has one additional *N-*methyltransferase gene (*abeM2*) that is not present in the *cla* cluster. Correspondingly, the cladoniamides lack one methyl group when compared to BE-54017 and its derivatives (Figure 2). Overall, the similarity of the gene clusters suggests that cladoniamide and BE-54017 derive from highly related biosynthetic pathways. Genes in the *cla* cluster have been named according to the conventions established in the naming of the genes in the *abe* gene cluster (Table 1) [13].

**Table 1:** Enzymes encoded by the cladoniamide gene cluster.

Enzyme	Size^a^	Deduced function	BE-54017homolog^b^	Size^a^	% ID^c^	Rebeccamycin homolog^d^	Size^a^	% ID^c^
ClaH	513	Tryptophan 5-chlorinase	AbeH	515	74	RebH	530	40
ClaT	422	Na+/H+ antiporter	AbeT	412	55	RebT	473	14
ClaF	177	Flavin reductase	AbeF	160	60	RebF	170	46
ClaY	278	α/β hydrolase	AbeY	264	56	-	-	-
ClaO	519	l-tryptophan oxidase	AbeO	513	66	RebO	473	51
ClaC	561	Flavin-dependent oxygenase	AbeC	533	69	RebC	529	59
ClaP	420	Cytochrome P450	AbeP	392	64	RebP	397	54
ClaX1	553	Flavin-dependent oxygenase	AbeX1	533	71	-	-	-
ClaM1	243	*N-*Methyltransferase	AbeM1	231	73	-	-	-
ClaD	1090	Chromopyrrolic acid synthase	AbeD	1015	68	RebD	1013	53
ClaX2	421	Flavin-dependent oxygenase	AbeX2	407	73	-		
ClaM3	344	*O*-methyltransferase	AbeM3	336	66	-		
ClaR	981	Transcriptional regulator	AbeR	932	46	RebR	923	35

a in amino acids.

b from GenBank accession JF439215 [13].

c percent identity on the amino acid level as determined by pair-wise ClustalW alignment [46].

d from GenBank accession AJ414559 [10].

### Gene cluster architecture

**Figure 3:**
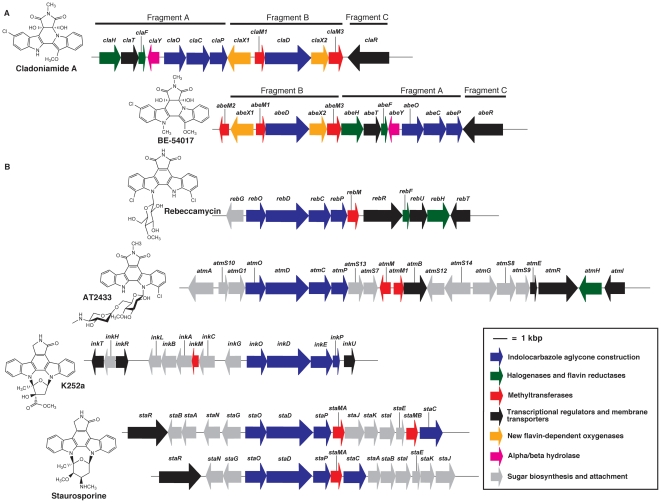
Comparison of indenotryptoline and indolocarbazole biosynthetic gene clusters. (A) Indenotryptoline gene clusters. Top to bottom: Cladoniamide and BE-54017 [13] gene clusters. (B) Indolocarbazole gene clusters. Top to bottom: rebeccamycin [10], AT2433 [16], K252a [17], staurosporine (from *Streptomyces* sp. TP-A0274 and putative cluster from *Streptomyces clavuligerus*) [9], [19], and staurosporine (putative cluster from *Salinispora arenicola*) [15] gene clusters. Note that *inkE* is the homolog to *rebC* in the K252a gene cluster, and the *Salinispora arenicola* staurosporine gene cluster lacks a *staMB* homolog.

The gene organization differs between the cladoniamide and the BE-54017 gene clusters. For the purpose of comparative analysis, the entire *cla* cluster can be considered to be composed of three fragments of DNA (Figure 3A). Fragment ‘A’ consists of *claH*, *claT*, *claF*, *claY*, *claO*, *claC*, and *claP.* Fragment ‘B’ consists of *claX1*, *claM1*, *claD*, *claX2*, and *claM3*. Finally, fragment ‘C’ is *claR*. These fragments have a different organization in the *abe* cluster where the order is Fragment B (*abeX1*, *abeM1*, *abeD*, *abeX2*, and *abeM3*), Fragment A (*abeH*, *abeT*, *abeF*, *abeY*, *abeO*, *abeC*, and *abeP*), and Fragment C (*abeR*), with *abeM2* appended upstream of Fragment B. This gene reorganization suggests that if the gene clusters were transferred between ancestral bacterial strains, a transposition of Fragment A occurred.

The *cla* cluster, from a *Streptomyces* strain, is 74% G+C, whereas the *abe* cluster, from an unknown source, is 71% G+C. High G+C content is a characteristic feature of actinomycete [18], and it is likely that the *abe* cluster, like the *cla* cluster, is derived from an actinomycete strain.

### Cladoniamides are likely to derive from an indolocarbazole precursor

Although the cladoniamides and BE-54017 have a unique chemical skeleton relative to other known bis-indole alkaloids, their biosynthetic gene clusters contain genes encoding enzymes needed for the construction of the more common bis-indolic scaffold, indolo[2,3-*a*]pyrrolo[3,4-*c*]carbazole. All of the characterized indolo[2,3-*a*]pyrrolo[3,4-*c*]carbazole gene clusters, including rebeccamycin (*reb*) [10], [11], AT2433 (*atm*) [16], K252a (*ink*) [17], and staurosporine (*sta*) [9], [15], [19] have four conserved genes (Figure 3B). These are genes encoding an l-tryptophan oxidase (*rebO* homolog), a chromopyrrolic acid synthase (*rebD* homolog), a cytochrome P450 (*rebP* homolog), and a flavin-dependent monooxygenase (*rebC* homolog). These four encoded enzymes are thought to react in sequence to generate the indolo[2,3-*a*]pyrrolo[3,4-*c*]carbazole scaffold from two molecules of l-tryptophan and molecular oxygen [12]. The presence of homologs of these four genes in both the cladoniamide and the BE-54017 gene clusters suggests that in both pathways the indolo[2,3-*a*]pyrrolo[3,4-*c*]carbazole scaffold is generated on the route to the final indenotryptoline products.

**Figure 4:**
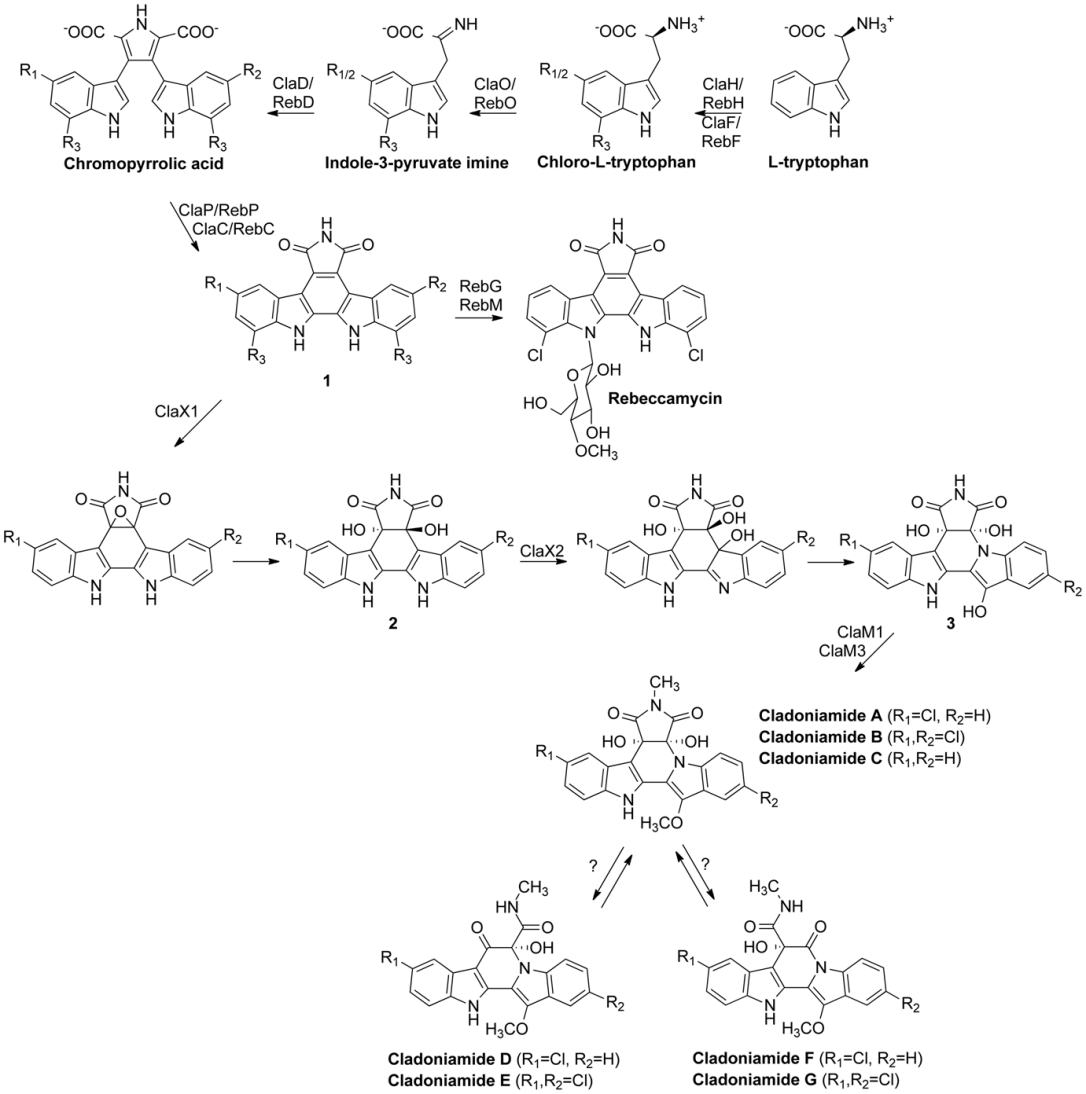
Postulated biosynthetic route to the cladoniamides and similarities to biosynthetic route to rebeccamycin. RebH, RebF, RebO, RebD, RebP, RebC, RebG, and RebM are enzymes from the rebeccamycin biosynthetic pathway (see Table 1) [10]. R_1_,R_2_ = H, R_3_ = Cl in rebeccamycin biosynthesis; R_1_ = Cl, R_2_,R_3_ = H in cladoniamide A, D, and F biosynthesis; R_1_,R_2_ = Cl, R_3_ = H in cladoniamide B, E, and G biosynthesis; R_1_,R_2_,R_3_ = H in cladoniamide C biosynthesis.

Two new flavin-dependent oxygenases are predicted in both the cladoniamide and BE-54017 biosynthetic pathways. Transposon mutagenesis of one of these, *abeX1* in the heterologously expressed BE-54017 gene cluster, eliminates BE-54017 production and leads to the accumulation of the indolo[2,3-*a*]pyrrolo[3,4-*c*]carbazole scaffold **1** (R_1_ = Cl, R_2_,R_3_ = H) (Figure 4), demonstrating that **1** is the likely substrate for AbeX1 [13]. Disruption of the cladoniamide homolog *claX1* is also anticipated to give accumulation of **1** because the encoded enzymes AbeX1 and ClaX1 are 71% identical. The presence of *abeX1* and *claX1* in both gene clusters strongly supports the role of an indolo[2,3-*a*]pyrrolo[3,4-*c*]carbazole intermediate in the biosynthesis of indenotryptolines.

Although biosynthetic construction via an indolocarbazole intermediate appears to be conserved between indolocarbazole and indenotryptoline pathways, the core *rebODCP* genes have been reorganized. In the rebeccamycin [10], AT2433 [16], and K252a [17] gene clusters the corresponding homologs, *rebODCP*, are clustered. In the staurosporine gene clusters found in *Streptomyces* sp. TP-A0274 [9], *Streptomyces clavuligerus* [19], and *Salinispora arenicola* [15], the *staODP* genes are similarly located together, but the *staC* gene is moved downstream. However, in both the *cla* and *abe* [13] clusters, a different local arrangement is observed in that the *rebD* homologs (*claD* and *abeD*) are separated from the *rebOCP* homologs (*claOCP* and *abeOCP*) (Figure 3).

### New enzymes in the indenotryptoline biosynthetic pathways

There are three enzymes unique to the indenotryptoline biosynthetic pathways. These are two putative flavin-dependent oxygenases (ClaX1 / AbeX1 and ClaX2 / AbeX2) and a putative α/β hydrolase (ClaY / AbeY). Comparative bioinformatics analysis and the genetic studies carried out on the *abe* cluster [13] provide considerable insight into the likely roles of the encoded enzymes. The two flavin-dependent oxygenases are described directly below, and discussion on the unknown role of the α/β hydrolase follows the description of the halogenase, flavin reductase, transcriptional regulator, membrane transporter, and methyltransferases identified in the *cla* cluster.

### Oxygenase ClaX1

A transposon mutant of *abeX1* in the BE-54017 heterologous expression system accumulates **1** (R_1_ = Cl, R_2_,R_3_ = H) and a transposon mutant of *abeX2* accumulates a methylated derivative of **2** (R_1_,R_2_ = H) (Figure 4) [13]. This finding suggests that the likely substrate of ClaX1 (71% identity to AbeX1) is **1** and that the enzyme is likely to install two hydroxyl groups during catalysis to give **2** (Figure 4), the likely substrate of ClaX2 (73% identity to AbeX2).

Further insight into the role of ClaX1 comes from consideration of related proteins, all of which are putative flavin-dependent oxygenases. The closest characterized homologs to ClaX1 are OxyL (31% identity) from the anhydrotetracycline biosynthetic pathway [20] and SsfO2 (29% identity) from the tetracycline SF2575 biosynthetic pathway [21]. These functionally equivalent enzymes are flavin-dependent dioxygenases that are thought to install two oxygen atoms on two carbon atoms on opposite sides of an electron rich tetracycline substrate via two independent flavin-dependent hydroxylation reactions (Figure S2A). However, a similar mechanism for double flavin-based hydroxylation chemistry of **1** is not possible because the carbon atoms targeted for hydroxylation lack hydrogen atoms that can be removed in the catalytic cycle (Figure S2A–C). Instead, an epoxidation of the double bond, followed by hydrolysis, would install two hydroxyl groups to give two tertiary alcohols. Indeed, other characterized homologs to ClaX1 are MtmOII [22] (30% identity) and TcmG [23] (26% identity). While the precise mechanisms of these latter enzymes are still under investigation, it is thought that both act as flavin-dependent epoxidases. An epoxidation mechanism is sensible for ClaX1's putative substrate **1** and gives the suspected product **2** after epoxide hydrolysis (Figure S2D). While it is likely that ClaX1 catalyzes the epoxidation, it is unknown if subsequent hydrolysis to give **2** is spontaneous or enzyme-catalyzed.

### Oxygenase ClaX2

A transposon mutant of *abeX2* in the BE-54017 heterologous expression system leads to accumulation of a methylated derivative of **2** (R_1_,R_2_ = H). **2** is the postulated substrate for AbeX2 [13]. It is likely that an equivalent result would be obtained for a deletion mutant of *claX2*, as ClaX2 is 73% identical to AbeX2. The route to generate downstream metabolites, e.g. indenotryptoline-containing molecules such as **3**, from this substrate **2**, via the action of a flavin oxygenase such as ClaX2, is not resolved. A chemically reasonable mechanism, as proposed by Williams et al. [3] and Chang and Brady [13], is epoxidation across a double bond. Cleavage of the epoxide could be driven through ketone formation from one tertiary alcohol, causing sigma-bond rupture and epoxide hydrolysis, opening the indolocarbazole scaffold. This cleaved molecule could then close through attack on the ketone by the indolic nitrogen, restoring the tertiary alcohol and arriving at the indenotryptoline scaffold **3** (Figure S3A).

A related mechanism is suggested through bioinformatics analysis. The closest characterized homolog to ClaX2 is RemO (45% identity) from the resistoflavin biosynthetic pathway. This flavin-dependent enzyme catalyzes a single hydroxylation on the *re* face of resistomycin, causing loss of aromaticity to yield resistoflavin (Figure S3B) [24]. A mechanism for ClaX2/AbeX2 catalysis more consistent with the known role of the close homolog RemO is the single hydroxylation on the 3′ position of one indole ring, causing loss in the aromaticity of the substrate. Further chemistry, as described above, including sigma-bond rupture to restore aromaticity to the indole ring, could lead to cleavage of the indolocarbazole. Then the ring could close via attack on the ketone by the indolic nitrogen, giving **3** (Figure S3C). Further biochemical characterization of ClaX2 will resolve whether ClaX2 is an epoxidase or a hydroxylase, and if subsequent steps are spontaneous or enzyme-mediated.

### Halogenase ClaH and flavin reductase ClaF

Both the *cla* and *abe* clusters encode a putative FADH_2_-dependent l-tryptophan chlorinase and an associated flavin reductase. These proteins are ClaH / AbeH (chlorinases) and ClaF / AbeF (reductases). The chlorinases have highest sequence identity to one another (74% identical) and to a number of established l-tryptophan chlorinases, including KtzR (65% identical to ClaH) and KtzQ (65% identity) from the kutzneride biosynthetic pathway [25], [26], PyrH (61% identity) from pyrroindomycin biosynthesis [27], RebH (40% identity) from rebeccamycin biosynthesis [10], [28], and PrnA (40% identity) from pyrrolnitrin biosynthesis [29], [30].

An *abeH* deficient mutant in the BE-54017 heterologous expression system accumulates the non-chlorinated BE-54017 derivative [13] and demonstrates that AbeH is a chlorinase. By extension, deletion of *claH* in *Streptomyces uncialis* would likely accumulate cladoniamide C, the non-chlorinated derivative of the major metabolite cladoniamide A. However, the stage in the biosynthetic pathway at which chlorine is added is not known: chlorine could be installed early in the pathway, with non-chlorinated substrates also accepted by all downstream enzymes, or the chlorine could be installed late in the pathway. Both chlorinated and non-chlorinated cladoniamides and BE-54017 derivatives are observed in extracts of *Streptomyces uncialis* and the heterologous expression system of BE-54017, respectively.

Given that AbeH and ClaH are highly related to characterized l-tryptophan chlorinases, and given that the chlorine is installed on the l-tryptophan substrate by RebH in the first step of the related rebeccamycin pathway, with all downstream enzymes also accepting non-chlorinated substrates [12], it is likely that chlorine is also installed early in the cladoniamide biosynthetic pathway, at the 5′ position on the l-tryptophan substrate, with downstream enzymes accepting both chlorinated and non-chlorinated substrates.

### Methyltransferases ClaM1 and ClaM3

The cladoniamides have two methyl groups in their structures, whereas BE-54017 and its derivatives have three methyl groups. Correspondingly, the *cla* cluster contains two methyltransferase genes (*claM1* and *claM3*), whereas the *abe* cluster contains three methyltransferase genes. *claM1* has highest similarity to *abeM1*. The encoded enzymes are 73% identical. Genetic studies show that *abeM1* encodes an *N*-methyltransferase that installs a methyl group on the succinimide nitrogen [13]. Both ClaM1 and AbeM1 also have moderate sequence identity (20% identity for ClaM1) with the corresponding *N*-methyltransferase AtmM1 from AT2433 biosynthesis [16]. This enzyme catalyzes the transfer of a methyl group to the succinimide nitrogen. *claM3* has highest similarity to *abeM3* (encoded enzymes are 66% identical), and genetic studies show that *abeM3* encodes an *O*-methyltransferase that installs a methyl group on the hydroxyl group [13]. ClaM3 also has high identity (36%) with the *O-*methyltransferase PokMT3 from polyketomycin biosynthesis [31], ElmNII from elloramycin biosynthesis (32% identity) [32], FdmN from fredricamycin biosynthesis (35% identity) [33], and the C-terminal domain of TcmN from tetracenomycin D3 biosynthesis (30% identity). Each of these enzymes is thought to catalyze the transfer of a methyl group to a phenolic oxygen, consistent with the likely role of ClaM3 in cladoniamide biosynthesis of installing a methyl group on the appended hydroxyl group. *abeM2*, which encodes the *N*–methyltransferase that installs a methyl group on the indole nitrogen of the tryptoline in BE-54017 biosynthesis, lacks a homolog in the cladoniamide biosynthetic gene cluster. Correspondingly, the cladoniamides lack methylation on the indole nitrogen of the tryptoline.

### Transcriptional regulator ClaR and membrane transporter ClaT

Both the *cla* and *abe* clusters contain genes encoding a membrane transporter and a transcriptional regulator. Among all published protein sequences, the transcriptional regulator ClaR found in the cladoniamide biosynthetic pathway has highest similarity to the corresponding transcriptional regulators from indolocarbazole biosynthetic pathways. This finding suggests that the pathways are all regulated through similar mechanisms. The transcriptional regulators with highest similarity are those from the published staurosporine biosynthetic gene clusters, including StaR from *Streptomyces* sp. TP-A0274 (35% identity), StaR from *Streptomyces clavuligerus* (34% identity), and StaR from *Salinispora arenicola* (Sare_2326; 33% identity). The protein also shares high similarity with the transcriptional regulator RebR from the rebeccamycin pathway (35% identity) and AtmR from the AT2433 pathway (34% identity). Like RebR, ClaR is a putative member of the Large ATP-binding regulators of the LuxR (LAL) family [10], [34].

ClaT is a putative membrane transporter, predicted to contain 11 transmembrane helices. Bioinformatics prediction suggests that it is a Na^+^/H^+^ antiporter, sharing moderate sequence similarity with the putative Na^+^/H^+^ antiporters AtmB from the AT2433 biosynthetic pathway (27% identity) [17] and RebT from the rebeccamycin biosynthetic pathway (14% identity) [10].

### Unknown role for the α/β hydrolase

The putative α/β hydrolase AbeY is not essential to the biosynthesis of BE-54017 or its derivatives in a heterologous expression system. Nonetheless, new low molecular weight metabolites (identities not reported) appeared in culture broths of *abeY* transposon mutants [13]. Given the close identity of *abeY* and *claY* (encoded enzymes are 56% identical), it is likely that an identical result would be seen for a *claY* deletion mutant in *Streptomyces uncialis*. As suggested by Chang and Brady, it is possible that another gene from the heterologous host *Streptomyces albus* complemented the *abeY* mutation and/or AbeY plays a role as modulating the activity of other enzymes. Further characterization of AbeY and ClaY is therefore warranted.

The likely role of these enzymes is unclear from bioinformatics analysis. The three closest homologs of AbeY and ClaY are annotated from genome sequencing projects but are not characterized. Possible roles for an α/β hydrolase in the biosynthesis of indenotryptolines are numerous. Enzymes with this type of fold catalyze ester hydrolysis, epoxide hydrolysis, amide bond hydrolysis, and other reactions [35], [36]. Since no enzyme is identified for the opening of the *N*-methylsuccinimide to give rise to the minor compounds, i.e. cladoniamides D-G and the ‘opened’ BE-54017 derivatives (Figure 2), it is tantalizing to suggest that ClaY/AbeY catalyzes the hydrolysis of an amide bond in the *N*-methylsuccinimide ring, which is followed by oxidative decarboxylation. In fact, there is no other candidate in the genes sequenced to date that could catalyze these reactions: likely roles for all enzymes in the pathways are now assigned, and heterologous expression of the BE-54017 gene cluster shows production of both ‘closed’ and ‘opened’ BE-54017 derivatives, showing that all enzymatic machinery is present in the heterologously expressed gene cluster.

An alternative explanation for the production of cladoniamides D–G and the ‘opened’ BE-54017 derivatives (Figure 2), which are minor compounds in the two series, is that hydrolysis and oxidative decarboxylation of the *N*-methylsuccinimide is spontaneous. However, no instability of either cladoniamide A or BE-54017 has been reported, and none of the related indolocarbazole compounds demonstrate spontaneous opening of the *N-*methylsuccinimide ring. Further biochemical studies will reveal if the α/β hydrolase ClaY plays a role in this reaction, in another reaction, or is entirely dispensable to the pathway.

### Likely biosynthetic route to the cladoniamides

An overall biosynthetic scheme to account for the construction of the indenotryptoline cores is shown in Figure 4. Although the order of chlorinations and methylations relative to other biosynthetic steps is currently unknown, this scheme is drawn to be consistent with the known pathway to rebeccamycin, where the chlorination occurs first [37] and the methylation occurs at the end of the biosynthesis [38], [39]. First, l-tryptophan is chlorinated at the 5′ position by the action of ClaH and a partner flavin reductase ClaF [28]. Next, 5-chloro-l-tryptophan reacts with ClaO, an amino acid oxidase [37], generating an indole-3-pyruvate imine. ClaD dimerizes two of these molecules to generate a chromopyrrolic acid molecule [40]. This molecule is in turn the substrate for ClaP, a cytochrome P450 enzyme, and ClaC, a flavin monooxygenase, which react in tandem to generate **1** [14]. Next, ClaX1 installs an epoxide over a double bond, followed by a spontaneous or enzyme-mediated epoxide opening to generate **2**. ClaX2 reacts to install a hydroxyl group or an epoxide, followed by spontaneous or enzyme-mediated chemistry to generate **3**. Methyltranferase reactions install methyl groups, with ClaM1 installing the methyl group on the nitrogen of the succinimide ring and ClaM3 installing a methyl group on the appended hydroxyl group. In the BE-54017 pathway, a methyl group would additionally be installed on the indolic nitrogen. Finally, the minor compounds cladoniamides D–G are generated through the opening of the *N*-methylsuccinimide ring and oxidative decarboxylation.

### Conclusion

This report of the cladoniamide gene cluster, together with the BE-54017 gene cluster and its genetic analysis [13], provides insight into the biosynthesis of the indenotryptoline scaffold from two molecules of l-tryptophan. Collectively, these results suggest that indenotryptolines are generated from the oxidative rearrangement of an indolopyrrolocarbazole scaffold via the action of two flavin-dependent enzymes, together with spontaneous chemistry. This work sets the stage for future biochemical, structural, and combinatorial biosynthetic studies of these new bis-indole biosynthetic pathways.

## Materials and Methods

### Materials

The cladoniamide producer *Streptomyces uncialis* was kindly provided by Julian E. Davis. Chemicals were purchased from Fisher Scientific Canada, except where noted otherwise below.

### Purification of genomic DNA

A mycelium glycerol stock of *Streptomyces uncialis* was streaked on ISP4 media (Difco) and grown at 30°C. A single colony was used to inoculate 2 mL of Tryptic Soy Broth medium (Difco) and grown at 30°C at 200 rpm for 5 days. This starter culture (1 mL) was used to inoculate 50 mL of YEME media (3 g/L yeast extract, 5 g/L bacto-peptone, 3 g/L malt extract, 10 g/L glucose, 340 g/L sucrose, 5 mM MgCl_2_), which was grown at 30°C at 200 rpm for 2 weeks. DNA was extracted via standard methods [41]. Specifically, cells were pelleted at 8000 rpm and washed in 10 mL of TE25S buffer (25 mM Tris-HCl pH 8, 25 mM EDTA, 0.3 M sucrose) three times. The pellet was resuspended in lysis buffer (TES25S supplemented with 3 mg/mL lysozyme from Sigma and 100 ng/mL RNAse from BioBasic Inc.) and incubated with frequent inversions at 37°C for 2 h. Proteinase K (100 µL of 20 mg/mL) and 1 mL of 10% SDS were added and the mixture was incubated at 55°C for 1 h. The mixture was placed on ice for 10 min, and 2.5 mL of 5 M potassium acetate was added and mixed by inversion. Phenol/chloroform/isoamyl alcohol (25∶24∶1) pH 8.05 (Invitrogen) (8 mL) was added and mixed by inversion for 6 min. This mixture was centrifuged for 15 min at 5000 x g at 4°C and the supernatant was transferred to a new tube using wide bore tips. The phenol/chloroform/isoamyl alcohol extraction step was repeated. Chloroform (8 mL) was added, followed by gentle inversion for 8 min and centrifugation. The supernatant was transferred to a new tube. Isopropanol (0.6 V) was added and mixed by inversion. Purified DNA was spooled using a sterile, flame-sealed Pasteur glass pipette. DNA was rinsed with 1 mL of 70% ethanol, air dried for 10 s, and dissolved overnight in TE (10 mM Tris pH 8.0, 1 mM EDTA) at 4°C.

### Design of primers for PCR-based library screening

A series of degenerate primers were designed to amplify indolocarbazole biosynthetic genes. Primers were purchased from Integrated DNA Technologies, Inc. The successfully used degenerate primer set, specific to the indolocarbazole biosynthetic gene *rebC* and its homologs (Figure S1), is RebC-degen-FP (5′-TCGGBCCSCGSTCSATGGA-3′) and RebC-degen-RP (5′-GCRCGGAASAGSAYGTTSCGRAA-3′) where R = A,G; Y = C,T; S = C,G; and B = C,G,T. This primer set was used to amplify genomic DNA from *Streptomyces uncialis* via PCR with a reaction mixture consisting of 1x EconoTaq buffer with MgCl_2_ (Lucigen), 5% DMSO, 250 µM dNTPs, 2.5 µM of each RebC-degen-FP and RebC-degen-RP, 0.8 ng/µL genomic DNA, and 2 U Taq polymerase in a temperature cycle of 95°C for 2 min; 30 cycles of 95°C for 30 s, gradient of 50–70°C for 45 s, 72°C for 2 min; 72°C for 10 min; 4°C hold. Successful amplification of a >400 basepair fragment was observed, as determined via ethidium bromide stained agarose gel electrophoresis, from reactions with extension temperatures between 53°C and 61°C. This fragment was purified from the PCR reactions according to the manufacturer's instructions using the GenElute PCR Clean-up kit (Sigma) and submitted for sequencing (Nucleic Acid Protein Service Unit, Michael Smith Laboratories, University of British Columbia) using the primer RebC-degen-FP. The resulting sequencing data (440 base pairs) was used for design of non-degenerate PCR primers specific to this sequencing data. These primers are RebC-uncial-FP (5′-GGACTGCGTCTGGGTCAC-3′) and RebC-uncial-RP (5′-CTCGATACCGGCGTCCTT-3′).

### Library construction and screening

Purified genomic DNA from *Streptomyces uncialis* (70 µg) was serially digested with *Sau*3A1 in 1x NEBuffer 4 (New England Biolabs) and 100 µg/mL bovine serum albumin (New England Biolabs). Appropriately digested genomic DNA fractions, as assayed by ethidium bromide staining of an agarose gel, were pooled and purified by phenol/chloroform/isoamyl alcohol extraction, chloroform extraction, and ethanol precipitation. FastAP (4 µL) (Fermentas) and 1x FastAP buffer (Fermentas) were added and the mixture was incubated at 37°C for 2 h. DNA was purified again by phenol/chloroform/isoamyl alcohol extraction, chloroform extraction, and ethanol precipitation. Separately, 16 µg of Supercos1 (Stratagene) was digested with 100 U of *Xba*I (New England Biolabs) in 1x NEBuffer 3 (New England Biolabs) and 100 µg/mL bovine serum albumin (New England Biolabs) for 3 h at 37°C. Enzyme was deactivated at 65°C for 20 min. FastAP (4 µL) (Fermentas) was added at 37°C for 3 h followed by enzyme deactivation at 70°C for 20 min. Finally, *Bam*HI (125 U) (New England Biolabs) was added, and the mixture was incubated at 37°C overnight. DNA was purified by phenol/chloroform/isoamyl alcohol extraction, chloroform extraction, and ethanol precipitation.

Digested genomic DNA (2.5 µg), 1 µg of digested Supercos1, 2.0 µL of 10x T4 DNA ligase buffer (New England Biolabs) and 1 µL of T4 DNA ligase (400,000 cohesive end units/ml; New England Biolabs) were incubated in a 20 µL volume at 4°C for 2 d. Ligations were titered using Max Planx Lambda Packaging Extract (Epicentre). Packaging extract (25 µL) was incubated with 10 µL of ligation mixture at 30°C for 90 min, 25 µL of additional packaging extract was added, followed by an additional incubation at 30°C for 90 min. Phage dilution buffer (10 mM Tris HCl, 100 mM NaCl, 10 mM MgCl_2_) (500 µL) was added, followed by gentle vortexing, and 25 µL of chloroform was added followed by gentle vortexing. Mixtures were again diluted 1∶1 in phage dilution buffer. XL1-MRF′ Blue Cells (Stratagene) were separately grown in LB + 10 mM MgSO_4_ + 0.2 % maltose from single colonies to an OD_600_ of ∼0.7. Cells were centrifuged at 500 g for 10 min and resuspended in an equal volume of 10 mM MgSO_4_ and incubated on ice. Cells (384 µL) were incubated with 16 µL of the diluted phage mixture and incubated at room temperature for 30 min. LB (800 µL) was added and the tubes were incubated at 37°C for 1 hour with gentle mixing every 15 min. 150 µL of this mixture was plated on each of six LB-agar-kanamycin sulfate (50 µg/mL) plates, and plates were incubated overnight at 37°C. Single colonies were picked from plates using toothpicks and used to inoculate individual wells of sixteen 96-well plates containing 150 µL of LB-kanamycin sulfate (50 µg/mL) per well and incubated overnight at 37°C.

Colonies from individual plates, individual rows, and individual columns were each pooled, glycerol was added, and plates were frozen at −80°C. Pools of colonies from each plate were individually analyzed via PCR using a reaction mixture consisting of 1x Lucigen EconoTaq buffer with MgCl_2_, 5% DMSO, 250 µM dNTPs, 250 nM of each RebC-uncial-FP and RebC-uncial-RP, 2 U Taq polymerase, and 2 µL of the pooled plates in a reaction volume of 25 µL. Reactions were cycled at 95°C for 2 min; 30 cycles of 95°C for 30 s, 66°C for 45 s, 72°C for 2 min; 72°C for 10 min; 4°C hold. Reactions were analyzed via ethidium bromide stained agarose gel electrophoresis. Plates that contained a *rebC* homolog by this PCR-based screening were subjected to further PCR-based screening to identify individual cosmids containing the *rebC* homolog. Five individual cosmids were identified that contained the *rebC* homolog via PCR-based screening. These cosmids were purified via standard methods and subjected to end sequencing using T3 and T7 primers (Nucleic Acid Protein Service Unit, Michael Smith Laboratories, University of British Columbia). One cosmid was chosen for total sequencing.

### Cosmid sequencing and annotation

The cosmid containing the cladoniamide gene cluster was sequenced via primer walking with at least double-stranded sequencing coverage at the Nucleic Acid Protein Service Unit at the Michael Smith Laboratories at the University of British Columbia. Primers were purchased from Integrated DNA Technologies, Inc. The DNA sequence was assembled and annotated using Sequencher (www.genecodes.com), FramePlot (http://www.nih.go.jp/fjun/cgi-bin/frameplot.pl) [42], and NCBI Blast (blast.ncbi.nlm.nih.gov/Blast.cgi) [43]. Transmembrane helices in ClaT were predicted using the TMHMM Server v. 2.0 (http://www.cbs.dtu.dk/services/TMHMM/) [44].

This sequence is available through GenBank (accession code JN165773).

## Supporting Information

Figure S1 **Partial sequence alignment of DNA encoding select** ***rebC*** **homologs.** *staC-1* is the *staC* gene from *Streptomyces* sp. TP-A0274 (GenBank accession AB088119.1) [9], *staC-2* is the *staC* gene from *Streptomyces longisporoflavus* (accession DQ861905.1) [14], *staC-3* is the putative *staC* gene from *Salinispora arenicola* (accession CP000850.1; Sare_2333) [15], *inkE* is from *Nonomuraea longicatena* (accession DQ399653.1) [17], *rebC* is from *Lechevalieria aerocolonigenes* (accession AJ414559.1) [10], and *atC* is from *Actinomadura melliaura* (accession DQ297453.1) [16]. Only DNA from 1 to ∼640 basepairs is shown. Highlighted in yellow are the DNA sequences targeted by the degenerate primers.
(PDF)

Figure S2 **Reaction of ClaX1.** (A) The closest characterized homologs to ClaX1 and AbeX1 are SsfO2 and OxyL. SsfO2 and OxyL react with 6-methyl-pretetramid through two rounds of flavin-based hydroxylation chemistry to give 4-keto-anhydrotetracycline [20], [21]. (B) The overall mechanism for the best-studied flavin-dependent hydroxylase, *para-*hydroxybenzoate hydroxylase (*p*HBH) is shown. After flavin-based chemistry occurs, the non-aromatic intermediate undergoes a rapid keto-enol tautomerization to restore aromaticity to the ring [45]. (C) If intermediate **1** underwent a single round of flavin-based hydroxylation chemistry to install a first hydroxyl group as shown, restoration of aromaticity following the reaction is not possible because the hydroxy group is on a carbon (starred) that lacks hydrogens. Without hydrogens, a tautomerization analogous to that shown for *p*HBH is not possible. (D) An epoxidation of **1**, followed by epoxide hydrolysis, would give rise to the proposed product **2**, containing two tertiary alcohols, and is consistent with the better established roles of MtmOII and TcmG.
(TIF)

Figure S3 **Reaction of ClaX2.** (A) One possible reaction mechanism for ClaX2 catalysis is epoxidation of **2** followed by sigma-bond cleavage and rearrangment of the indolocarbazole to give the indenotryptoline core structure **3**. (B) The closest characterized homolog to ClaX2/AbeX2 is RemO, which catalyzes a single hydroxylation of a multi-ringed aromatic substrate on the *re* face [24]. (C) Similar flavin-based hydroxylation chemistry on **2** could lead to an intermediate that undergoes sigma-bond cleavage and rearrangment of the indolocarbazole to give the indenotryptoline core **3**.
(TIF)
