# Supplementary material for: Biosynthetic Gene Cluster for the Cladoniamides, Bis-Indoles with a Rearranged Scaffold
Source: PLoS One. 2011 Aug 18;6(8):e23694. doi: 10.1371/journal.pone.0023694 (PMC3158105; doi:10.1371/journal.pone.0023694)
Supplement: Figure S1 — Partial sequence alignment of DNA encoding select rebC homologs. staC-1 is the staC gene from Streptomyces sp. TP-A0274 (GenBank accession AB088119.1) [9], staC-2 is the staC gene from Streptomyces longisporoflavus (accession DQ861905.1) [14], staC-3 is the putative staC gene from Salinispora arenicola (accession CP000850.1; Sare_2333) [15], inkE is from Nonomuraea longicatena (accession DQ399653.1) [17], rebC is from Lechevalieria aerocolonigenes (accession AJ414559.1) [10], and atC is from Actinomadura melliaura (accession DQ297453.1) [16]. Only DNA from 1 to ∼640 basepairs is shown. Highlighted in yellow are the DNA sequences targeted by the degenerate primers. (PDF) [file pone.0023694.s001.pdf]

```
staC-1          ATGACGCATTCCGGTGAGCGGACCGATGTGCTGATCGTGGGCGGCGGCCCGGTCGGGATG 60
staC-2          ATGACGCATTCCGGTGAGCGGACCGATGTGCTGATCGTGGGCGGCGGCCCGGTCGGGATG 60
staC-3          ATGACAAACTCCGGCGAGTGGACCGACGTGCTGATCGTGGGCGGCGGCCCGGTCGGGATG 60
inkE            ATGAC---TCGCAGCGAAGAGACCGACGTCCTCATCGTGGGCGGCGGCCCCGTGGGCCTG 57
rebC            ATGAACGCGCCCATCGA---AACAGACGTGCTGATCCTGGGCGGAGGTCCGGTGGGCATG 57
atC             ATGACCACGGCTTACGA---GACCGACGTCCTGGTCATCGGCGGCGGCCCGGTTGGCATG 57
                ****           **    ** ** ** **  ** * ***** ** ** ** **  **

staC-1          GCGCTGGCGCTGGATCTGAGGTACCGGGGAATCGACTGTCTGGTCGTCGACGCCGGTGAC 120
staC-2          GCGCTGGCGCTGGATCTGAGGTACCGGGGCATCGACTGTCTGGTCGTCGACGCCGGTGAC 120
staC-3          GCGTTGGCACTGGACCTGAGGTACCGCGGAATCGACTGCATGGTTGTCGAAGCGGGCGAC 120
inkE            GCCCTGTCACTGGACCTCACCCATCGCGGCGTCCGGCACATCGTGATCGACGCGGGCGAC 117
rebC            GCGCTGGCGCTCGACCTCGCCCATCGCCAGGTCGGCCACCTCGTCGTGGAGCAGACCGAC 117
atC             GCGCTGGTGCTGGACCTGAAGTACCGGGGAGTCGGCTGCCAGCTCATCGAGGCGAGCGAC 117
                **  **   ** ** **     * **     **          *  * **       ***

staC-1          GGCACGGTCCGGCACCCCAAGGTCAGCACCATCGGTCCCCGCTCGATGGAACTCTTCCGC 180
staC-2          GGCACGGTCCGGCACCCCAAGGTCAGCACCATCGGTCCCCGCTCGATGGAACTCTTCCGC 180
staC-3          GGCGAGGTCCGGCACCCCAAGGTGAGTACGATCGGCCCGCGCTCGATGGAGCTGTTTCGC 180
inkE            GGCGTGGTGCGCCACCCGAAGGTCAGCACGGTCGGGCCGCGCTCGATGGAGCACTTCCGC 177
rebC            GGTACGATCACCCACCCGCGGGTCGGCACCATCGGCCCGCGGTCCATGGAACTCTTCCGG 177
atC             GGCTCGGTCTCGCATCCGCGGGTGGGCTCGATCGGTCCCCGGTCGATGGAGCTGTTCCGC 177
                **   * *    ** **   ***  *  *  **** ** ** ** ***** *  ** **

staC-1          CGCTGGGGCACCGC-GGACGCCATCCGGAACGCCGGCTGGCCCGCCGACCATCCCCTGGA 239
staC-2          CGCTGGGGCGCCGC-GGACGCGATCCGGAACGCCGGCTGGCCCGCCGACCATCCCCTGGA 239
staC-3          CGTTGGGGTCTCGC-GGACACCATCCGTAACGCGGGATGGCCCGCCGACCATCCCCTGGA 239
inkE            CGCTGGGGGGTGGCCGGGCG-GGTCAGGGACGCGGGCTGGCCGCCCGGTCACCCGCTCGA 236
rebC            CGCTGGGGTGTCGCGAAGCA-GATCCGCACCGCCGGGTGGCCCGGCGACCATCCGCTCGA 236
atC             AGGTGGGGGATCGCCGACCG-CATCCGTGCCGCCGGCTGGCCCGGCGACCACTCGCTCGA 236
                 * *****    **    *    ** *   *** ** *****   **  **  * ** **

staC-1          CATCGCCTGGGTGACCAAGGTCGGCGGCCACGAGATCTACCGCTACCGGCGGGGCACGGC 299
staC-2          CATCGCCTGGGTGACCAAGGTCGGCGGCCACGAGATCTACCGCTACCGGCGAGGCACGGC 299
staC-3          CATCGCCTGGGTCACCAGAGTCGGTGGCCACGAGGTGCACCGGTATCGGCGGGGCACGAC 299
inkE            CATCGCGTGGGTGACCCGCGTCGGCGAGTACGAGATCCACCGGTTCGAGCGCGGCACCGC 296
rebC            CGCCGCGTGGGTGACGAGGGTGGGCGGCCACGAGGTGTACAGAATCCCGCTCGGCACCGC 296
atC             CACCGCCTGGGTCACTCAGGTGGGCGGGCACGAGATCCACCGGCTCCGGGTGGGCACCGC 296
                *  *** ***** **    ** ** *   ***** *  ** *      *   *****  *

staC-1          CGCGAACCGCCCGGCCTTCGTCCATACCCCCGAGCCCGATCAGATCTGCCCCGCCCACTG 359
staC-2          CGCGAACCGCCCGGCCTTCGTCCATACCCCCGAGCCCGATCAGATCTGCCCCGCCCACTG 359
staC-3          GGCGAACCGTGGGCCCTACGTACACACCCCTGAGCCCGACCAGATCTGTCCGGCGCACTG 359
inkE            CGCCGCCCGCCCGGTGTTCCGCCACACGCCGGAGCCGGACCAGGTGTGCCCGGCCCACCT 356
rebC            GGACACCAGGGCGACACCCGAGCACACACCAGAACCCGACGCGATCTGCCCGCAGCACTG 356
atC             CGACACCAGGCCGCTGCCGCCGTACACGCCGGAACCGGAACAGGTCTGCCCGCAGCACTG 356
                 *    * *   *          * ** ** ** ** **   * * ** **    ***

staC-1          GCTCAACCCGGTGCTG-ATCGAGGCCGTGGGCGTCCACCCGGACGGCCCCCTGCG-ACTG 417
staC-2          GCTCAACCCGGTGCTG-ATCGAGGCCGTGGGCGTCCACCCGGACGGCCCCCTGCT-ACTG 417
staC-3          GCTGAATCCACTGTTGCAACG-GGCCGTCGGCGTGCACCCCACCGGTCCACTGCG-GCTC 417
inkE            GCTGAACCCGGTGCTG----GCGGGCG-CGGTGGGCA---GGCCGCTGCGCTAC---CT- 404
rebC            GCTCGCACCCCTGCTG-GCGGAGG----CCGT--------CGGCGAGAGGCTGCGCACCC 403
atC             GCTGGCGCCCCTGCTG-CTGGAGGAGGCCCGCACCCACCCCGGCGGGGTCGTGCGCACCC 415
                ***    **  ** **    * **      *            **      * *   *

staC-1          AGCACGACCGTCGACGGCGTGGTCCAGACCGACGACCACGTCGAGGCCAC--CCTCACCG 475
staC-2          AGTACGACCGTCGACGGCGTGGTCCAGACCGACGACCACGTCGAGGCCAC--CCTCACCG 475
staC-3          AGGACGACCGTGGACCGCGTGCGTCAGGCGGCCGACCATGTCGAGGCCAC--CCTCGTCG 475
inkE            -GCACCGCC-TGGAGCACTTCGAGCAGGACGCCGGTTGCGTGCGGGCCACGATCTCCTCG 462
rebC            GCTCGCGGC-TGGACTCCTTCGAGCAGCGCGACGACCACGTCCGCGCCACG--ATCACCG 460
atC             GGTGCCGAC-TCGACGGCTTCACCCAGCACGACGACCACGTCGAGGCGACG--GTCACCG 472
                        * * **   * *    ***   * **     **    ** **    **  **
```

```
staC-1          ACC--ACGCCACCGGCACCACCGGCACCGTCCGGGCACGCTTCCTGGTCGCCTGTGACGG 533
staC-2          ACC--ACGCCACCGGCACCACCGGCACCGTCCGGGCACGCTTCCTCGTCGCCTGTGACGG 533
staC-3          AGG--ACGGTTCCGGAAGGACCGGCACCGTCCGCGCACAATTCCTGGTTGCCTGCGACGG 533
inkE            A----ACGGC----GAGGAATCGGT--CGTCTGGGCGCGCTATCTGGTGGCCTGCGACGG 512
rebC            ACCT--CCGCACGGGTGCCACCCGTGCCGTGCACGCCAGGTATCTGGTGGCGTGTGACGG 518
atC             ACCTGGCCG-AGGGGCGCGAGCTCCGG-ATCCGCGCCCGCTACATGGTGGCGGTGGACGG 530
                *     *       *    * *       *    **    *   * ** **    *****

staC-1          CGCCTCCTCGCCCATCCGCCGGGCCTGCGGCATCGAGGCACCGGCCCGCCAC-CGTACG- 591
staC-2          CGCCTCCTCGCCCGTCCGCCGGGCCTGCGGCATCGAGGCACCGGCCCGCCAC-CGTACG- 591
staC-3          GGCTGCCTCACCCATTCGGCAGGCCTGCGGCATCGACGCACCGCCACGCCAC-ACGACG- 591
inkE            CAGCTCCTCCGTCGTGCGCAAGGCCCTCGGCATCGACTCGCCCGCCCGCCAC-GAGGCG- 570
rebC            CGCCTCCTCCCCCACCCGCAAGGCCCTCGGCATCGACGCGCCACCGAGGCAC-AGGACG- 576
atC             CGCGTCCTCTCCGGTGCGCAAGGCCTGCGGCATC---CCGTCGTCGGCTCGCTACGACGT 587
                     ****       **  ****   *******    *  *  *    * *     **

staC-1          CA-GGTCTTCCGCAACATCCTCTTCCGCGCCCCCGAACT---CAAGGACC------GCCT 641
staC-2          CA-GGTCTTCCGCAACATCCTCTTCCGCGCCCCCGAGCT---CAAGGACC------GCCT 641
staC-3          CA-GGTCTTTCGGAACATCCTCTTCCGTGCCCCGGAACT---CAAGCGGC------AACT 641
inkE            CA-GGTCTTCCGCAACGTGCTCTTCCGCGCGCCGGGGCTGCCCGCGCGCCTCGCGGAGCG 629
rebC            CA-GGTGTTCCGCAACATCCTGTTCCGCGCCCCCGAACTG--CGTTCGCT-------CCT 626
atC             CATGACCTTCCGCAACATCCTCTTCCGTGCGCCGGAGCTG--CGTGCGCG-------GCT 638
                ** *   ** ** *** * ** ***** ** ** *  **   *               *
```
