# Supplementary figures and images for: Biosynthetic Gene Cluster for the Cladoniamides, Bis-Indoles with a Rearranged Scaffold
Source: PLoS One. 2011 Aug 18;6(8):e23694. doi: 10.1371/journal.pone.0023694 (PMC3158105; doi:10.1371/journal.pone.0023694)

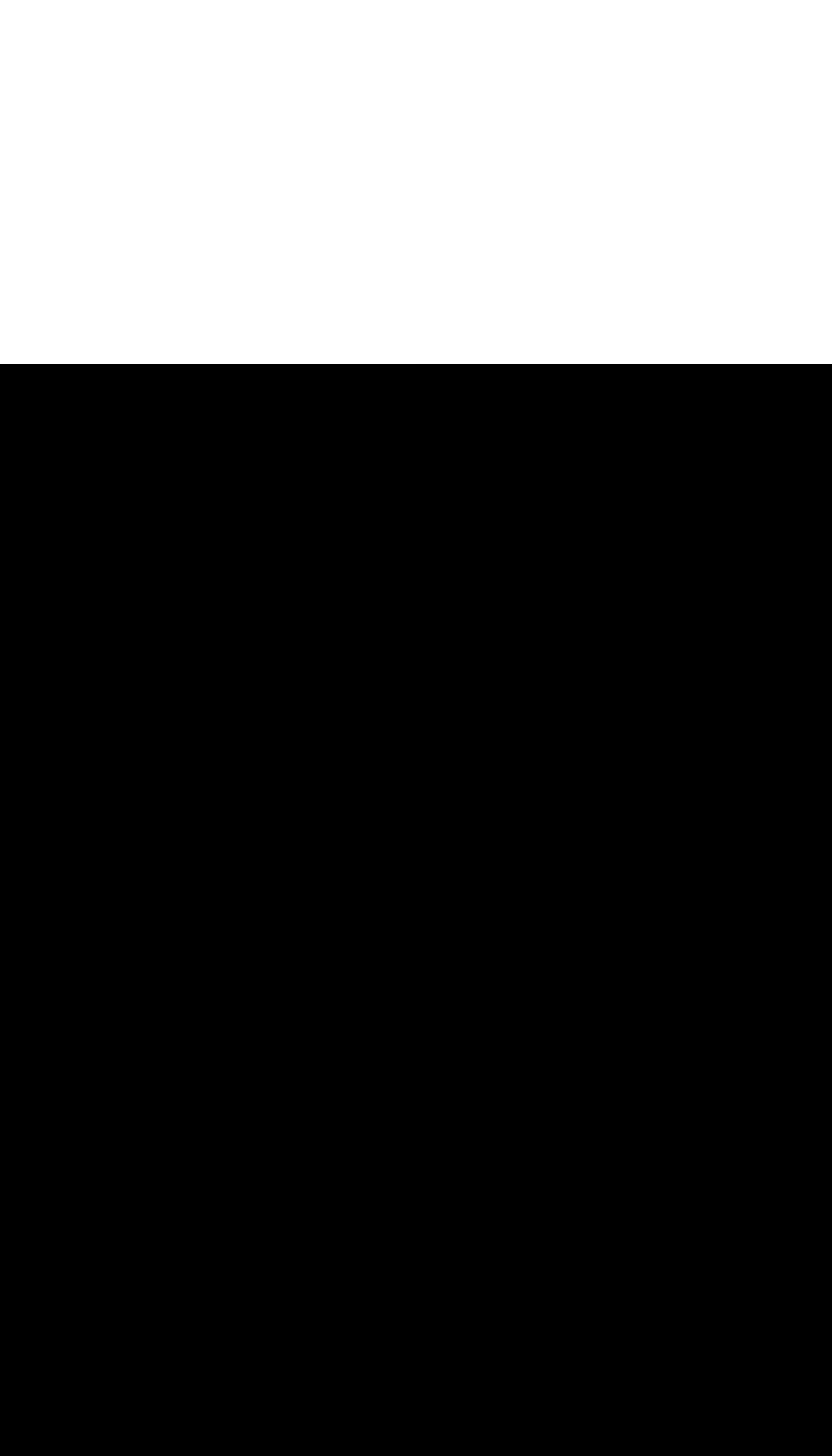

Supplement: Figure S3 — Reaction of ClaX2. (A) One possible reaction mechanism for ClaX2 catalysis is epoxidation of 2 followed by sigma-bond cleavage and rearrangment of the indolocarbazole to give the indenotryptoline core structure 3. (B) The closest characterized homolog to ClaX2/AbeX2 is RemO, which catalyzes a single hydroxylation of a multi-ringed aromatic substrate on the re face [24]. (C) Similar flavin-based hydroxylation chemistry on 2 could lead to an intermediate that undergoes sigma-bond cleavage and rearrangment of the indolocarbazole to give the indenotryptoline core 3. (TIF) [file pone.0023694.s003.tif]
